# Preferences for Starting Daily, On-Demand, and Long-Acting Injectable HIV Preexposure Prophylaxis Among Men Who Have Sex With Men in the United States (2021-2022): Nationwide Online Cross-Sectional Study

**DOI:** 10.2196/62801

**Published:** 2024-11-13

**Authors:** Duygu Islek, Travis Sanchez, Jennifer L Glick, Jeb Jones, Keith Rawlings, Supriya Sarkar, Patrick S Sullivan, Vani Vannappagari

**Affiliations:** 1 Rollins School of Public Health Emory University Atlanta, GA United States; 2 Community Health Science & Policy (CHSP) LSUHSC New Orleans, LA United States; 3 ViiV Healthcare Durham, NC United States

**Keywords:** preexposure prophylaxis, men who have sex with men, gay, HIV, HIV prevention, United States, long-acting, injectable, sociodemographic, illicit drug use, adherence, sexually transmitted infection, reproductive health, sexual behavior, HIV treatment

## Abstract

**Background:**

Long-acting (LA) injectable preexposure prophylaxis (PrEP) and on-demand PrEP may improve overall PrEP uptake among men who have sex with men (MSM), but little is understood about the PrEP option preferences of MSM in practical scenarios where they may choose between various PrEP options.

**Objective:**

This study aims to examine the preferences for starting various PrEP options among a US nationwide online convenience sample of MSM from September 2021 to February 2022.

**Methods:**

Participants reporting no prior HIV diagnosis were provided brief descriptions of each PrEP option and were asked, “If [PrEP option] were available from your local doctor and you could access it for free, would you go to your doctor in the next month to start [PrEP option]?” Those who said “yes” to multiple options were asked to rank them in order of preference. MSM currently taking daily oral (DO) PrEP were asked whether they would switch to on-demand or LA PrEP options. Log binomial models were created to examine the association between willingness to start or switch to on-demand and LA PrEP with various sociodemographic and behavioral factors.

**Results:**

In the analytic sample (N=7760), among the participants who did not use any PrEP in the past 12 months (n=5108, 66%), 54% (n=2445) reported willingness to start at least 1 PrEP option and 41% (n=1845) of participants showed interest in starting multiple PrEP options. Overall, the highest willingness was reported for on-demand PrEP (n=2235, 44%), followed by DO PrEP (n=2174, 43%) and LA PrEP (n=1482, 29%). LA PrEP was ranked first among those interested in multiple options. Characteristics associated with ranking LA PrEP as a first option to start PrEP versus DO or on-demand PrEP were region of residence (residing in the West vs Northeast), report of sexually transmitted infection diagnosis in the past year, report of illicit drug use other than marijuana in the past year, and prior awareness of LA PrEP. Among current DO PrEP users (n=2379, 31%), 58% (n=1386) were willing to switch to on-demand or LA PrEP, and LA PrEP was ranked first among participants who were open to switching to both options. Willingness to switch to LA PrEP was higher among those who used illicit drugs other than marijuana in the past year, who heard of LA PrEP prior to the survey, and those who took 15 or less doses of oral PrEP in the last 30 days.

**Conclusions:**

LA PrEP was the highest-ranked option among most MSM who were willing to try multiple options or switch from DO PrEP. These findings highlight that LA PrEP might fill coverage gaps among MSM who use illicit drugs, have had a recent sexually transmitted infection diagnosis, and have less than optimal DO PrEP adherence.

## Introduction

Daily oral (DO) HIV preexposure prophylaxis (PrEP) effectively prevents HIV acquisition when taken as directed [1]; however, PrEP initiation and adherence are still low among men who have sex with men (MSM) in the United States [2]. Also, many studies report that MSM who start PrEP have low adherence to DO pills [3,4] which results in less protection from HIV acquisition, as adherence is a key factor for effective protection of PrEP [5].

Alternative PrEP options, such as long-acting (LA) injectable and oral on-demand PrEP, may improve PrEP uptake. LA PrEP is approved by the US Food and Drug Administration [6] and has proven to be more effective than DO PrEP in preventing HIV acquisition among MSM [7]. LA PrEP only necessitates a single injection every 2 months, which may result in fewer concerns about adherence among MSM who initiate PrEP or switch from DO to LA PrEP, ultimately leading to better effectiveness [8,9]. LA PrEP could also potentially provide more confidentiality than daily oral PrEP and reduce stigma among MSM [10]. The use of on-demand PrEP, in which individuals align their pill-taking schedule with periods of sexual activity, is reported to decrease HIV transmission risk, despite that it is not a US Food and Drug Administration–approved or recommended dosing schedule by the Centers for Disease Control and Prevention [11-13]. On-demand PrEP could potentially enhance PrEP uptake and adherence, particularly for MSM who are worried about side effects or who have difficulty consistently taking daily pills or prefer to take fewer pills overall [14-17]. These PrEP options offer an opportunity to increase PrEP initiation and adherence. However, there is limited information about the PrEP option preferences of MSM in practical scenarios where they may choose or switch between various PrEP options. Understanding MSM’s preferences and ranking of PrEP options could inform health care provider–patient discussions in clinical settings.

Some sociobehavioral factors could be associated with relative preferences of a particular PrEP regimen among MSM. For example, racial differences in willingness to use LA PrEP were previously reported [18,19]. Also, young age [20], geographical residence [21], and health insurance coverage [22] might shape relative preferences for PrEP initiation among MSM. Behavioral factors, such as the number of partners, unprotected sex [23], history of sexually transmitted infections [24], and illicit drug use [25] could affect patients’ preferences for a particular PrEP regimen. However, little is known about how these sociodemographic and behavioral factors impact patient PrEP option preferences. We aimed to examine relative preferences for starting PrEP regimens and their associations with sociodemographic and behavioral characteristics among a US nationwide online convenience sample of MSM.

## Methods

### Study Design and Analytic Sample

The American Men’s Internet Survey (AMIS) collects data annually from MSM via a self-administered online survey. The AMIS investigation team recruits participants through English and Spanish-language banner advertisements placed on websites and social networking sites used by MSM as well as by email blasts on LGBTQ+ (lesbian, gay, bisexual, transgender, queer/questioning, and others)-specific email listservs. The advertisements are displayed online based on self-reported demographic information related to online profiles. Participants who click an advertisement or use an email survey link are taken to the eligibility screener. Participants who are eligible for the survey are then taken to the online informed consent page. After participants provide consent, they enter responses to survey questions directly into their own computer, tablet, or smartphone, via a web interface. The online survey includes questions on demographics, sexual behaviors, substance use, HIV and STI testing and diagnosis, and use of HIV prevention services [26,27]. For this analysis, we used data from the 2021 AMIS data collection cycle which were collected from September 2021 to February 2022. Participants were eligible to participate in AMIS if they were aged 15 years or older, were assigned male sex at birth and reported current male sex identity, resided in the United States and provided a US ZIP code, and reported having ever had oral or anal sex or both with a male partner at least once or identified as gay or bisexual if they were in the age group of 15-17 years. For this analysis, additional eligibility criteria were having had oral or anal sex with another man in the past 12 months and no self-reported prior HIV diagnosis.

### Outcome Measures

Participants were first given a brief description of each PrEP option: DO, on-demand, and LA PrEP (Table S1 in Multimedia Appendix 1). To determine willingness to start each PrEP option, participants were asked “If [PrEP option] were available from your local doctor and you could access it for free, would you go to your doctor in the next month to start [PrEP option]?” Based on the responses, willingness to use the PrEP option was grouped as “willing to start PrEP option” and “not willing to use PrEP option or not sure.” Current DO PrEP users among the analytic sample were determined by asking “Are you currently taking PrEP?” Participants who currently use DO PrEP were asked about their willingness to switch to on-demand or LA PrEP by asking “If [PrEP option] were available from your local doctor and you could access it for free, would you go to your doctor in the next month to start [PrEP option]?” Participants who had discontinued DO PrEP in the past 12 months (ie, those who used DO PrEP in the past 12 months but were not current users, n=261) were not asked willingness questions. If participants were willing to start multiple PrEP options, they were then asked to rank the PrEP options by preference.

### Covariate Measures

Sociodemographic characteristics were age (15-24, 25-29, 30-39, and 40 years and older), race or ethnicity (Non-Hispanic or Latino Black, Hispanic or Latino, Non-Hispanic or Latino White, and other or multiple racial groups), health insurance type (private, public, other or multiple insurances, and no insurance), county of residence urbanicity based on the National Center for Health Statistics urban-rural classification scheme for counties (large central metro, large fringe metro, medium metro, small metro, micropolitan, and noncore) [28], and census region (Northeast, Midwest, South, and West).

Behavioral characteristics were self-reported for the past 12 months for condomless anal sex with a male partner (yes or no), number of male sex partners (one, or two or more), sexually transmitted infection (STI) diagnosis (yes or no), marijuana use (yes or no), and illicit drug use other than marijuana (yes or no).

To determine participants’ prior awareness of PrEP options, they were asked “Before today, have you ever heard of [PrEP option]?” If participants were current oral PrEP users, they were asked about their prescription medication brand (Truvada or Descovy), how many doses of oral PrEP they took in the last 30 days (15 or less doses, 16-29 doses, and 30 doses), and how many months in a row they have been taking oral PrEP (less than 2 months, 2-6 months, 7-12 months, and 12 or more months) to describe PrEP use characteristics.

### Statistical Analysis

We described the sociodemographic, behavioral, and PrEP use characteristics in the analytic sample. We report the distribution of willingness to start each PrEP option for participants who were or were not using PrEP at the time they completed the survey, overall and by participant characteristics. We examined the distribution of first preference for the PrEP option among participants willing to use multiple PrEP options. We used log-binomial regression models to examine the association of each characteristic with the willingness to start each PrEP option, using unadjusted and adjusted prevalence ratios. To estimate the adjusted prevalence ratios and 95% CI, we included the sociodemographic (age, race or ethnicity, health insurance, urbanicity, and census region), behavioral (condomless anal sex, number of male sex partners, STI diagnosis, marijuana use, and other illicit drug use), and PrEP option awareness variables in multivariable log-binomial regression models. We retained these variables in the multivariable models since they were shown to have associations with PrEP willingness in previous literature, despite the lack of significance in this univariate analysis. We followed the same approach for those who were currently using PrEP; however, we additionally included PrEP use characteristics (current PrEP prescription medication, number of PrEP doses taken in the last 30 days, and PrEP duration) in our multivariable models to estimate the adjusted prevalence ratios and 95% CIs among this group of participants.

LA PrEP was the first preference for PrEP modality among participants who were not currently on PrEP and were willing to use multiple PrEP regimens. To better understand this finding, we examined characteristics associated with ranking LA PrEP as the first preference versus DO or on-demand PrEP. Among participants who were not currently using oral PrEP, we combined those who reported willingness to use (1) DO and LA PrEP; (2) on-demand and LA PrEP; and (3) DO, on-demand, and LA PrEP and created univariate and multivariable log-binomial regression models to estimate unadjusted and adjusted PRs and 95% CIs. We also examined the characteristics associated with ranking LA PrEP as a first preference versus on-demand PrEP among current DO PrEP users. We used univariate log-binomial regression because a multivariable modeling approach was not possible due to small cell numbers and the distribution of covariates in this group. We conducted the data analysis with SAS version 9.4 (SAS Institute).

### Ethical Considerations

The core AMIS study was reviewed and approved by Emory University’s human subjects research ethics board (IRB00047676). Informed consent is collected from participants before their participation in the core AMIS study. Eligible participants were shown a consent form to review and asked whether they wished to participate in the AMIS survey. Participants who provided informed consent were taken to a screen where they could complete the online AMIS survey. Those who did not consent were taken to a screen thanking them for their interest and no further information was collected. Emory University has determined that this substudy does not comprise human subjects research because it only consists of secondary analysis. Therefore, no informed consent process is required for the substudy. Participants did not receive any compensation for their participation. The study data are nonidentifiable and do not pose a risk of loss of confidentiality.

## Results

In the analytic sample (N=7760), 45.2% (n=3511) of participants were aged 40 years and older, 14% (n=1086) were Hispanic or Latino, 10% (n=784) were non-Hispanic or Latino Black, and 66.6% (n=5114) were non-Hispanic or Latino White individuals (Table S2 in Multimedia Appendix 1). Most participants had private health insurance, had a college degree or postgraduate education, and were employed with full-time wages. Approximately 45% (n=3467) of the participants reported residing in large central metro areas; 38% (n=2931) resided in the South. 74% (n=5774) of the participants had condomless anal sex and 78% (n=6061) had two or more male sex partners in the past 12 months. 34% of the participants used DO PrEP in the past 12 months (n=2652). Among those who used PrEP in the past 12 months, 90% (n=2379) were currently using DO PrEP. Among those who did not use PrEP in the past 12 months (n=5108, 66%, 7% (n=334) reported ever taking daily oral PrEP.

**Figure 1:**
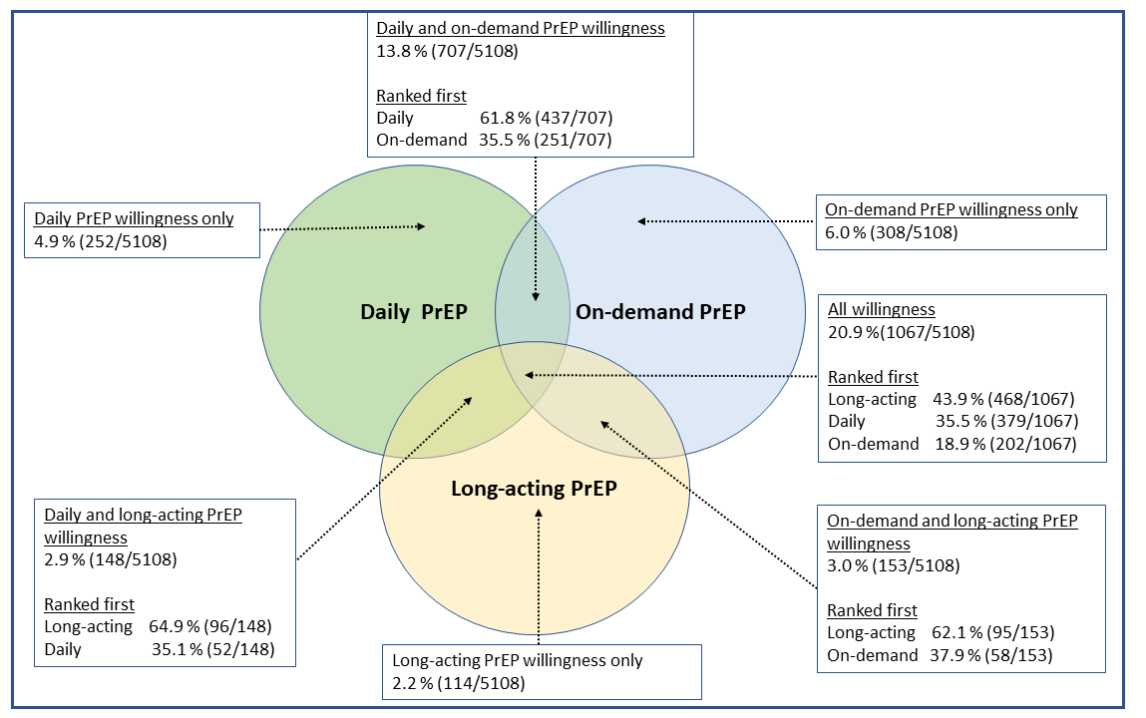
Willingness and relative preferences to start PrEP options among men who have sex with men who did not use PrEP in the past 12 months, American Men’s Internet Survey, 2021-2022. PrEP: preexposure prophylaxis.

Among participants who did not use any PrEP in the past 12 months (n=5108, 66%), 54% (n=2794) reported willingness to start at least 1 PrEP option. Overall, the highest willingness was reported for on-demand PrEP (n=2445, 44%), followed by DO PrEP (n=2174, 43%) and LA PrEP (n=1482, 29%). However, 40.6% (n=2075) of participants showed interest in starting multiple PrEP options and LA PrEP was ranked first among those interested in multiple options (Figure 1).

In multivariable modeling, willingness to start LA PrEP was significantly higher among Hispanic or Latino participants compared to White participants (Table 1). Participants who had an STI diagnosis, had condomless anal sex, had 2 or more male sex partners, and used illicit drugs other than marijuana in the past 12 months were significantly more willing to start LA PrEP. Prior awareness of LA PrEP was also associated with increased willingness to start LA PrEP.

In multivariable modeling, willingness to start on-demand PrEP was significantly higher among the youngest age group (15-24 years) compared to those who are 40 years and older and Hispanic or Latino participants compared to White participants (Table 2).

**Figure 2:**
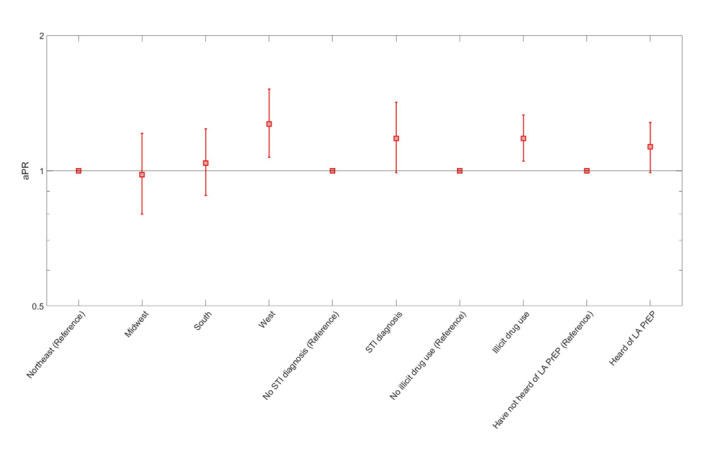
Characteristics associated with ranking long-acting injectable PrEP as a first preference to start PrEP versus daily oral or on-demand PrEP among men who have sex with men who did not use PrEP in past 12 months, American Men’s Internet Survey, 2021-2022. aPR: adjusted prevalence ratio; LA: long-acting; PrEP: preexposure prophylaxis; STI: sexually transmitted infection.

Participants who had an STI diagnosis, had condomless anal sex, and had 2 or more male sex partners in the past 12 months were significantly more willing to start on-demand PrEP. Characteristics associated with ranking LA PrEP as a first option to start PrEP versus DO or on-demand PrEP were region of residence (residing in the West vs Northeast), report of STI diagnosis (vs no diagnosis), report of illicit drug use (vs no drug use) and prior awareness of LA PrEP (vs not being aware of LA PrEP; Figure 2; and Table S3 in Multimedia Appendix 1).

**Figure 3:**
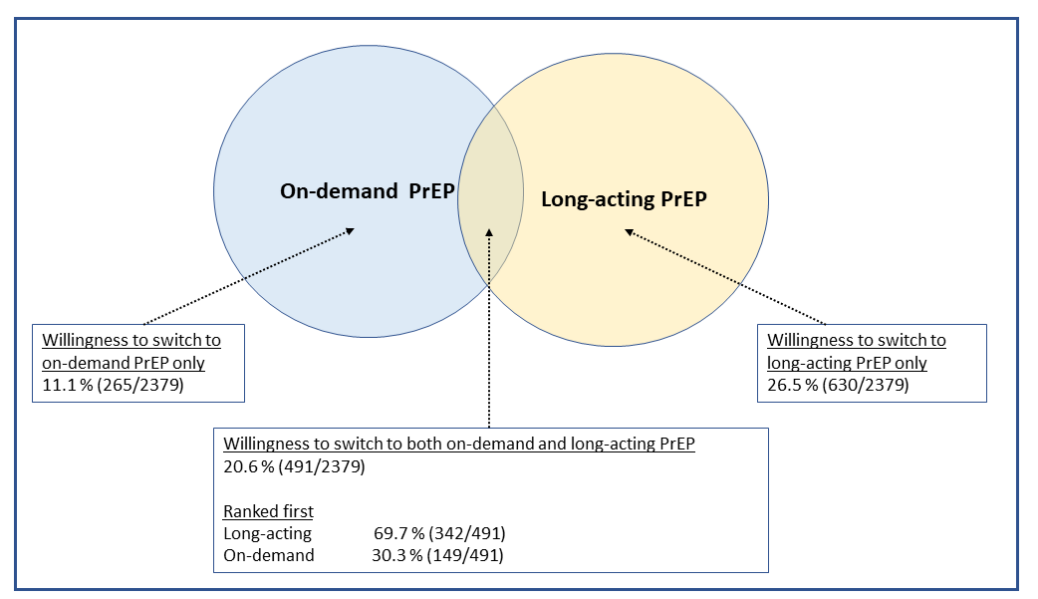
Willingness and relative preferences to switch to other PrEP options among men who have sex with men who are current daily oral PrEP users, American Men’s Internet Survey, 2021-2022. PrEP: preexposure prophylaxis.

Among participants who were currently using DO PrEP (n=2379, 31% of the whole analytic sample), 58% (n=1386) were willing to switch to on-demand or LA PrEP. Willingness to switch to LA PrEP (n=1121, 47.1%) was higher than willingness to switch to on-demand PrEP (n=756, 31.7%). LA PrEP was ranked first among participants who were open to switching to both options (Figure 3).

In multivariate modeling, there were no meaningful associations with sociodemographic characteristics and willingness to switch to LA PrEP among those currently using DO PrEP, except that willingness was slightly increased in Hispanic or Latino participants compared to White participants (Table 3).

Willingness to switch to LA PrEP was higher among those who used illicit drugs other than marijuana in the past 12 months and among those who had previously heard of LA PrEP. There were no meaningful associations between current PrEP prescription medication and PrEP duration with the willingness to switch to LA PrEP. Those who took 15 or less doses of DO PrEP and who took 16-29 doses in the last 30 days were more willing to switch to LA PrEP, compared to those who took all 30 doses**.**

In multivariable modeling, willingness to switch to on-demand PrEP was significantly higher among those who lived in rural micropolitan or noncore areas compared to those who lived in large central metro areas (Table 4).

**Table 1:** Willingness to use LA^a^ injectable PrEP^b^ among men who have sex with men who did not use oral PrEP in the past 12 months, American Men’s Internet Survey, 2021-2022 (n=5108).

			Willing to use LA PrEP (n=1482), n (%)^c^		Not willing to use LA PrEP or not sure (n=3063), n (%)^c^		Unadjusted PR^d^ (95% CI)		Adjusted PR (95% CI)^e^
**Age (years)**									
	15-24	172 (32.6)		356 (67.4)		1.05 (0.91-1.21)		1.03 (0.90-1.17)	
	25-29	169 (33.7)		333 (66.3)		1.05 (0.91-1.21)		1.04 (0.91-1.20)	
	30-39	383 (34.8)		719 (65.2)		1.11 (1.00-1.23)		1.02 (0.92-1.13)	
	40 and older	758 (31.4)		1655 (68.6)		Reference		Reference	
**Race or ethnicity**									
	Black, non-Hispanic or Latino	153 (34.4)		292 (65.6)		1.16 (1.01-1.34)		1.13 (0.98-1.30)	
	Hispanic or Latino	276 (42.9)		368 (57.1)		1.42 (1.27-1.58)		1.41 (1.26-1.57)	
	White, non-Hispanic or Latino	917 (30.3)		2113 (69.7)		Reference		Reference	
	Other or multiple races	123 (31.9)		263 (68.1)		1.04 (0.88-1.22)		1.06 (0.90-1.24)	
**Health insurance**									
	None	147 (40.2)		219 (59.8)		1.21 (1.05-1.39)		1.07 (0.93-1.23)	
	Private only	992 (32.4)		2069 (67.6)		Reference		Reference	
	Public only	246 (32.2)		517 (67.8)		0.99 (0.88-1.12)		0.99 (0.88-1.11)	
	Other or multiple insurances	70 (26.4)		195 (73.6)		0.83 (0.67-1.02)		0.86 (0.70-1.06)	
**NCHS^f^ urban-rural category**									
	Large central metro	561 (32.1)		1188 (67.9)		Reference		Reference	
	Large fringe metro	323 (31.9)		689 (68.1)		1.00 (0.89-1.12)		1.06 (0.94-1.18)	
	Medium metro	326 (34.5)		619 (65.5)		1.08 (0.96-1.21)		1.05 (0.93-1.19)	
	Small metro	126 (33.3)		252 (66.7)		1.06 (0.91-1.25)		1.02 (0.86-1.20)	
	Micropolitan and noncore	139 (31.2)		306 (68.8)		0.98 (0.83-1.15)		0.98 (0.83-1.16)	
**Census region**									
	Northeast	262 (31.4)		572 (68.6)		Reference		Reference	
	Midwest	277 (29.2)		673 (70.8)		0.90 (0.78-1.04)		0.91 (0.79-1.05)	
	South	610 (34.4)		1163 (65.6)		1.08 (0.95-1.22)		1.05 (0.93-1.19)	
	West	330 (33.8)		647 (66.2)		1.08 (0.94-1.24)		1.04 (0.91-1.18)	
**STI^g^ diagnosis in the past 12 months**									
	No	1369 (31.6)		2959 (68.4)		Reference		Reference	
	Yes	113 (52.1)		104 (47.9)		1.63 (1.41-1.87)		1.26 (1.10-1.45)	
**Condomless anal sex in the past 12 months**									
	No	405 (26.8)		1104 (73.2)		Reference		Reference	
	Yes	1077 (35.5)		1959 (64.5)		1.32 (1.20-1.46)		1.26 (1.14-1.39)	
**Number of male sex partners**									
	One	260 (20.5)		1007 (79.5)		Reference		Reference	
	Two or more	1191 (37.2)		2014 (62.8)		1.83 (1.62-2.06)		1.82 (1.62-2.06)	
**Marijuana use in the past 12 months**									
	No	1144 (32.0)		2429 (68.0)		Reference		Reference	
	Yes	338 (34.8)		634 (65.2)		1.09 (0.98-1.20)		0.89 (0.78-1.02)	
**Other illicit drug use past 12 months**									
	No	1182 (31.0)		2625 (69.0)		Reference		Reference	
	Yes	300 (40.7)		438 (59.3)		1.31 (1.19-1.45)		1.26 (1.10-1.44)	
**Prior awareness of LA PrEP**									
	No	1184 (31.5)		2569 (68.4)		Reference		Reference	
	Yes	292 (37.3)		490 (62.7)		1.20 (1.08-1.33)		1.22 (1.10-1.35)	

^a^LA: long-acting.

^b^PrEP: preexposure prophylaxis.

^c^Data does not add up to the total number of participants due to missing information resulting from non-response from some of the participants.

^d^PR: prevalence ratio.

^e^Log-binomial models are adjusted for age, race or ethnicity, health insurance, the NCHS rural-urban category, census region, STI diagnosis in the past 12 months, condomless anal sex in the past 12 months, number of male sex partners, marijuana use, other illicit drug use past 12 months (other than marijuana), prior awareness of LA PrEP.

^f^NCHS: National Center for Health Statistics.

^g^STI: sexually transmitted infection.

**Table 2:** Willingness to use on-demand PrEP^a^ among men who have sex with men who did not use PrEP in the past 12 months, American Men’s Internet Survey, 2021-2022 (n=5108).

			Willing to use on-demand PrEP (n=2235), n (%)^b^	Not willing to use on-demand PrEP or not sure (n=2355), n (%)^b^	Unadjusted PR^c^ (95% CI)	Adjusted PR (95% CI)^d^
**Age (years)**						
		15-24	291 (54.3)	245 (45.7)	1.12 (1.03-1.23)	1.13 (1.04-1.23)
		25-29	235 (46.5)	270 (53.5)	0.96 (0.87-1.07)	1.02 (0.92-1.12)
		30-39	529 (47.8)	578 (52.2)	0.99 (0.92-1.06)	1.00 (0.93-1.08)
		40 and older	1180 (48.3)	1262 (51.7)	Reference	Reference
**Race or ethnicity**						
		Black, non-Hispanic or Latino	228 (50.3)	225 (49.7)	1.08 (0.98-1.19)	1.01 (0.91-1.11)
		Hispanic or Latino	357 (54.9)	293 (45.1)	1.18 (1.09-1.27)	1.14 (1.05-1.24)
		White, non-Hispanic or Latino	1427 (46.7)	1628 (53.3)	Reference	Reference
		Other or multiple races	212 (54.2)	179 (45.8)	1.16 (1.05-1.28)	1.14 (1.03-1.25)
**Health insurance**						
		None	217 (58.8)	152 (41.2)	1.21 (1.10-1.33)	1.12 (1.02-1.22)
		Private only	1500 (48.5)	1591 (51.5)	Reference	Reference
		Public only	348 (45.1)	424 (54.9)	0.93 (0.85-1.01)	0.90 (0.83-0.99)
		Other or multiple insurances	132 (49.3)	136 (50.7)	1.02 (0.89-1.15)	1.02 (0.91-1.15)
**NCHS^e^ urban-rural category**						
		Large central metro	851 (48.2)	915 (51.8)	Reference	Reference
		Large fringe metro	491 (48.2)	527 (51.8)	1.00 (0.92-1.08)	1.04 (0.96-1.13)
		Medium metro	469 (49)	489 (51)	1.02 (0.94-1.10)	1.06 (0.97-1.16)
		Small metro	193 (50.7)	188 (49.3)	1.05 (0.94-1.17)	1.10 (0.98-1.23)
		Micropolitan and noncore	220 (48.8)	231 (51.2)	1.01 (0.91-1.13)	1.04 (0.93-1.16)
**Census region**						
		Northeast	408 (48.2)	439 (51.8)	Reference	Reference
		Midwest	437 (45.3)	527 (54.7)	0.94 (0.85-1.04)	0.94 (0.85-1.03)
		South	875 (49)	909 (51)	1.02 (0.94-1.11)	1.01 (0.93-1.09)
		West	508 (51.6)	476 (48.4)	1.07 (0.98-1.18)	1.06 (0.97-1.16)
**STI^f^ diagnosis in the past 12 months**						
		No	2093 (47.9)	2280 (52.1)	Reference	Reference
		Yes	142 (65.4)	75 (34.6)	1.37 (1.24-1.51)	1.19 (1.07-1.32)
**Condomless anal sex in the past 12 months**						
		No	689 (45.2)	837 (54.8)	Reference	Reference
		Yes	1546 (50.5)	1518 (49.5)	1.12 (1.05-1.19)	1.09 (1.02-1.17)
**Number of male sex partners**						
		One	403 (31.5)	876 (68.5)	Reference	Reference
		Two or more	1795 (55.5)	1442 (44.5)	1.76 (1.61-1.92)	1.75 (1.60-1.91)
**Marijuana use in the past 12 months**						
		No	1724 (47.7)	1891 (52.3)	Reference	Reference
		Yes	511 (52.4)	464 (47.6)	1.10 (1.03-1.18)	0.97 (0.89-1.07)
**Other illicit drug use past 12 months**						
		No	1816 (47.2)	2034 (52.8)	Reference	Reference
		Yes	419 (56.6)	321 (43.4)	1.20 (1.12-1.29)	1.11 (1.01-1.22)
**Prior awareness of on-demand PrEP**						
		No	1671 (48.8)	1754 (51.2)	Reference	Reference
	Yes		561 (48.3)	600 (51.7)	0.99 (0.92-1.06)	0.98 (0.91-1.04)

^a^PrEP: preexposure prophylaxis.

^b^Data does not add up to the total number of participants due to missing information resulting from non-response from some of the participants.

^c^PR: prevalence ratio.

^d^Log-binomial models are adjusted for age, race/ethnicity, health insurance, NCHS rural-urban category, census region, STI diagnosis in the past 12 months, condomless anal sex in the past 12 months, number of male sex partners, marijuana use, other illicit drug use past 12 months (other than marijuana), prior awareness of on-demand PrEP.

^e^NCHS: National Center for Health Statistics.

^f^STI: sexually transmitted infection.

**Table 3:** Willingness to switch to LA^a^ injectable PrEP^b^ among men who have sex with men who are current oral PrEP users, American Men’s Internet Survey, 2021-2022 (n=2379).

			Willing to switch to LA PrEP (n=1211), n (%)^c^		Not willing to switch to LA PrEP or not sure (n=1232), n (%)^c^		Unadjusted PR^d^ (95% CI)		Adjusted PR (95% CI)^e^
**Age (years)**									
	15-24	47 (49)		49 (51)		1.07 (0.86-1.32)		0.96 (0.76-1.21)	
	25-29	107 (46.5)		123 (53.5)		1.02 (0.87-1.18)		0.97 (0.82-1.13)	
	30-39	348 (51.5)		328 (48.5)		1.12 (1.02-1.23)		1.06 (0.96-1.17)	
	40 and older	619 (45.8)		732 (54.2)		Reference		Reference	
**Race or ethnicity**									
	Black, non-Hispanic or Latino	95 (66)		49 (34)		0.96 (0.82-1.13)		0.94 (0.80-1.11)	
	Hispanic or Latino	163 (57)		123 (43)		1.18 (1.06-1.33)		1.13 (1.00-1.28)	
	White, non-Hispanic or Latino	750 (69.6)		328 (30.4)		Reference		Reference	
	Other or multiple races	103 (12.3)		732 (87.7)		0.97 (0.83-1.13)		0.95 (0.81-1.12)	
**Health insurance**									
	None	48 (49.5)		49 (50.5)		1.03 (0.84-1.26)		0.94 (0.74-1.19)	
	Private only	896 (48.1)		965 (51.9)		Reference		Reference	
	Public only	126 (47)		142 (53)		0.98 (0.85-1.12)		0.99 (0.86-1.13)	
	Other or multiple insurances	44 (38.6)		70 (61.4)		0.80 (0.63-1.02)		0.74 (0.57-0.97)	
**NCHS^f^ urban-rural category**									
	Large central metro	630 (47.8)		687 (52.2)		Reference		Reference	
	Large fringe metro	210 (46.6)		241 (53.4)		0.97 (0.87-1.09)		1.01 (0.90-1.13)	
	Medium metro	179 (49.2)		185 (50.8)		1.03 (0.91-1.16)		1.04 (0.91-1.20)	
	Small metro	59 (49.2)		61 (50.8)		1.03 (0.85-1.24)		1.05 (0.85-1.30)	
	Micropolitan and noncore	42 (42)		58 (58)		0.88 (0.69-1.11)		0.93 (0.72-1.20)	
**Census region**									
	Northeast	222 (47.3)		247 (52.7)		Reference		Reference	
	Midwest	186 (44.2)		235 (55.8)		0.93 (0.81-1.08)		0.94 (0.81-1.09)	
	South	415 (49.4)		425 (50.6)		1.04 (0.93-1.17)		1.11 (0.98-1.25)	
	West	298 (47.8)		325 (52.2)		1.01 (0.89-1.15)		1.01 (0.89-1.15)	
**STI^g^ diagnosis in the past 12 months**									
	No	824 (46.7)		939 (53.3)		Reference		Reference	
	Yes	297 (50.3)		293 (49.7)		1.08 (0.98-1.18)		1.02 (0.92-1.13)	
**Condomless anal sex in the past 12 months**									
	No	108 (49.8)		109 (50.2)		Reference		Reference	
	Yes	1013 (47.4)		1123 (52.6)		0.95 (0.83-1.10)		0.94 (0.81-1.10)	
**Number of male sex partners**									
	One	42 (43.3)		55 (56.7)		Reference		Reference	
	2 or more	1029 (47.4)		1142 (52.6)		1.09 (0.87-1.38)		1.15 (0.90-1.48)	
**Marijuana use in the past 12 months**									
	No	721 (45.3)		872 (54.7)		Reference		Reference	
	Yes	400 (52.6)		360 (47.4)		1.16 (1.07-1.27)		1.00 (0.89-1.13)	
**Other illicit drug use past 12 months**									
	No	749 (44.7)		927 (55.3)		Reference		Reference	
	Yes	372 (54.9)		305 (45.1)		1.23 (1.13-1.34)		1.17 (1.03-1.32)	
**Prior awareness of LA PrEP**									
	No	690 (45)		843 (55)		Reference		Reference	
	Yes	429 (52.6)		387 (47.4)		1.17 (1.07-1.27)		1.17 (1.07-1.28)	
**Current PrEP prescription medication**									
	Truvada	613 (48.5)		650 (51.5)		1.05 (0.96-1.14)		1.05 (0.96-1.15)	
	Descovy	490 (46.2)		570 (53.8)		Reference		Reference	
**Number of PrEP doses taken in last 30 days**									
	15 or less	85 (57)		64 (43)		1.28 (1.10-1.48)		1.24 (1.05-1.48)	
	16-29	242 (58)		175 (42)		1.30 (1.18-1.43)		1.27 (1.14-1.40)	
	30	776 (44.6)		964 (55.4)		Reference		Reference	
**PrEP duration**									
	Less than 2 months	88 (49.4)		90 (50.6)		1.05 (0.90-1.23)		0.96 (0.80-1.15)	
	2 to 6 months	170 (50)		170 (50)		1.06 (0.94-1.19)		1.06 (0.94-1.21)	
	7 to 12 months	119 (46.7)		136 (53.3)		0.99 (0.86-1.14)		0.98 (0.85-1.13)	
	12 months or more	740 (47.2)		829 (52.8)		Reference		Reference	

^a^LA: long-acting.

^b^PrEP: preexposure prophylaxis.

^c^Data does not add up to the total number of participants due to missing information resulting from non-response from some of the participants

^d^PR: prevalence ratio.

^e^Log-binomial regression models are adjusted for age, race or ethnicity, health insurance, NCHS rural-urban category, census region, STI diagnosis in the past 12 months, condomless anal sex in the past 12 months, number of male sex partners, marijuana use, other illicit drug use past 12 months (other than marijuana), prior awareness of LA PrEP, current PrEP prescription medication, number of PrEP doses taken in the last 30 days, PrEP duration.

^f^NCHS: National Center for Health Statistics.

^g^STI: sexually transmitted infection.

**Table 4:** Willingness to switch to on-demand PrEP^a^ among men who have sex with men who are current oral PrEP users, American Men’s Internet Survey, 2021-2022 (n=2379).

		Willing to switch to on-demand PrEP (n=756), n (%)^b^	Not willing to switch to on-demand PrEP or not sure (n=1597), n (%)^b^	Unadjusted PR^c^ (95% CI)	Adjusted PR (95% CI)^d^
**Age (years)**					
	15-24	28 (29.2)	68 (70.8)	0.86 (0.62-1.18)	0.80 (0.58-1.12)
	25-29	70 (30.3)	161 (69.7)	0.89 (0.72-1.10)	0.84 (0.68-1.05)
	30-39	198 (29.3)	478 (70.7)	0.86 (0.75-0.99)	0.86 (0.74-1.00)
	40 and older	460 (34.1)	890 (65.9)	Reference	Reference
**Race or ethnicity**					
	Black, non-Hispanic or Latino	67 (31.9)	143 (68.1)	1.02 (0.83-1.26)	1.09 (0.88-1.36)
	Hispanic or Latino	102 (34.8)	191 (65.2)	1.11 (0.94-1.32)	1.14 (0.95-1.36)
	White, non-Hispanic or Latino	500 (31.3)	1096 (68.7)	Reference	Reference
	Other or multiple races	78 (34.5)	148 (65.5)	1.10 (0.91-1.34)	1.15 (0.95-1.39)
**Health insurance**					
	None	41 (42.3)	56 (57.7)	1.36 (1.07-1.73)	1.24 (0.97-1.59)
	Private only	580 (31.1)	1282 (68.9)	Reference	Reference
	Public only	98 (36.7)	169 (63.3)	1.18 (0.99-1.40)	1.11 (0.93-1.33)
	Other or multiple insurances	32 (28.1)	82 (71.9)	0.90 (0.67-1.22)	0.83 (0.61-1.12)
**NCHS^e^ urban-rural category**					
	Large central metro	397 (30.1)	922 (69.9)	Reference	Reference
	Large fringe metro	159 (35.3)	292 (64.7)	1.17 (1.01-1.36)	1.12 (0.96-1.31)
	Medium metro	119 (32.8)	244 (67.2)	1.09 (0.92-1.29)	1.11 (0.91-1.36)
	Small metro	40 (33.3)	80 (66.7)	1.11 (0.85-1.45)	1.01 (0.75-1.38)
	Micropolitan and noncore	41 (41.4)	58 (58.6)	1.38 (1.07-1.76)	1.39 (1.05-1.83)
**Census region**					
	Northeast	144 (30.7)	325 (69.3)	Reference	Reference
	Midwest	127 (30.2)	294 (69.8)	0.98 (0.81-1.20)	1.08 (0.88-1.32)
	South	285 (34)	554 (66)	1.11 (0.94-1.31)	1.13 (0.95-1.35)
	West	200 (32.1)	424 (67.9)	1.04 (0.87-1.25)	1.06 (0.88-1.27)
**STI^f^ diagnosis in the past 12 months**					
	No	597 (33.9)	1166 (66.1)	Reference	Reference
	Yes	159 (26.9)	431 (73.1)	0.80 (0.69-0.92)	0.85 (0.72-0.99)
**Condomless anal sex in past 12 months**					
	No	97 (44.9)	119 (55.1)	Reference	Reference
	Yes	659 (30.8)	1478 (69.2)	0.69 (0.58-0.81)	0.70 (0.60-0.83)
**Number of male sex partners**					
	One	39 (40.2)	58 (59.8)	Reference	Reference
	2 or more	701 (32.3)	1469 (67.7)	0.80 (0.63-1.03)	0.89 (0.69-1.16)
**Marijuana use in past 12 months**					
	No	515 (32.3)	1077 (67.7)	Reference	Reference
	Yes	241 (31.7)	520 (68.3)	0.98 (0.86-1.11)	0.99 (0.83-1.18)
**Other illicit drug use past 12 months**					
	No	539 (32.2)	1136 (67.8)	Reference	Reference
	Yes	217 (32)	461 (68)	0.99 (0.87-1.13)	1.11 (0.93-1.32)
**Prior awareness of LA PrEP**					
	No	388 (33.5)	770 (66.5)	Reference	Reference
	Yes	367 (30.8)	826 (69.2)	0.92 (0.82-1.03)	0.96 (0.85-1.08)
**Current PrEP prescription medication**					
	Truvada	397 (31.4)	867 (68.6)	Reference	Reference
	Descovy	348 (32.9)	711 (67.1)	1.05 (0.93-1.18)	1.01 (0.89-1.14)
**Number of PrEP doses taken in last 30 days**					
	15 or less	96 (64.4)	53 (35.6)	2.42 (2.10-2.79)	—^g^
	16-29	175 (42)	242 (58)	1.57 (1.37-1.81)	—
	30	464 (26.7)	1276 (73.3)	Reference	—
**PrEP duration**					
	Less than 2 months	77 (43.3)	101 (56.7)	1.50 (1.25-1.81)	1.53 (1.26-1.87)
	2 to 6 months	125 (36.8)	215 (63.2)	1.28 (1.09-1.50)	1.28 (1.08-1.50)
	7 to 12 months	97 (38.2)	157 (61.8)	1.33 (1.11-1.58)	1.31 (1.10-1.56)
	12 months or more	452 (28.8)	1118 (71.2)	Reference	Reference

^a^PrEP: preexposure prophylaxis.

^b^Data does not add up to the total number of participants due to missing information resulting from non-response from some of the participants.

^c^PR: prevalence ratio.

^d^Log-binomial regression models are adjusted for age, race or ethnicity, health insurance, NCHS rural-urban category, census region, STI diagnosis in the past 12 months, condomless anal sex in the past 12 months, number of male sex partners, marijuana use, other illicit drug use past 12 months (other than marijuana), prior awareness of on-demand PrEP, current PrEP prescription medication, PrEP duration.

^e^NCHS: National Center for Health Statistics.

^f^STI: sexually transmitted infection.

^g^Multivariate models did not converge when the “number of PrEP doses taken in the last 30 days” variable was included in the models. Therefore, we excluded this variable from multivariate models and we only report results from the univariate analysis.

Willingness to switch to on-demand PrEP was also significantly higher among those who had been using PrEP for less than 2 months compared to those who had used PrEP for more than 12 months. Despite a strong association between willingness to switch to on-demand PrEP and fewer doses of DO PrEP taken in the last 30 days in bivariate analyses, this association was not stable in multivariable analyses. Among 150 participants who took 15 or less doses in the last 30 days, 42% intended to take PrEP only when they had sex, and 10% intended to take PrEP on some other schedule.

Characteristics associated with ranking LA PrEP as a first option to switch to versus on-demand PrEP were other or multiple races (vs White, non-Hispanic or Latino), illicit drug use, and prior awareness of LA PrEP (Table S4 in Multimedia Appendix 1).

## Discussion

### Principal Findings

There was substantial interest in starting LA and on-demand PrEP among US MSM in our nationwide study. Among MSM who were not currently using DO PrEP, the highest willingness was reported for on-demand PrEP. However, a substantial proportion of MSM were willing to start multiple PrEP options, and LA PrEP was ranked first when those willing to start multiple types of PrEP were asked to make a single choice. More than half of US MSM in our study who were currently using DO PrEP expressed interest in switching to LA PrEP or on-demand PrEP; when both options were selected, they expressed preference toward LA PrEP versus on-demand PrEP. Multiple demographic factors, risk behaviors, and prior PrEP experiences were associated with these preferences.

Previous research has highlighted the potential benefits in coverage or uptake of adding LA PrEP as an additional PrEP option because many current PrEP users reported a preference toward LA PrEP over DO PrEP [29,30]. Additionally, prior studies have shown the potential for LA PrEP to increase PrEP uptake overall, as many individuals who could benefit from some form of PrEP preferred LA PrEP to DO PrEP [31,32]. Our findings build upon this existing literature demonstrate that LA PrEP is the highest-ranked option when participants are open to using multiple PrEP options or are willing to switch from daily oral PrEP.

Our findings also highlight that PrEP option preferences are not uniform for US MSM and vary by key characteristics. Hispanic or Latino MSM showed a significantly higher willingness to start or switch to LA PrEP and on-demand PrEP compared to White MSM. Younger MSM expressed a significantly higher willingness to start on-demand PrEP versus older MSM. These results align with previous studies that reported high PrEP willingness among Hispanic participants [18,33] and young MSM [34]. However, despite high PrEP willingness, PrEP uptake and adherence remain suboptimal in young and Hispanic MSM [2,35,36]. In particular, DO PrEP adherence was shown to be low among young MSM [37]. One of the difficulties that MSM often face is the requirement to consistently take their daily medication, which can be challenging to remember [38,39]. Challenges with adherence to daily pills might explain the higher willingness toward on-demand PrEP among young MSM in our study. Nevertheless, recent studies report that LA PrEP could expand access to PrEP for young MSM who are wary of adhering to DO or on-demand PrEP [39].

Willingness to start on-demand or LA PrEP was also associated with behavioral risk factors in our study, such as having a recent STI diagnosis, having two or more male sex partners, and having condomless anal sex. Recent studies estimating the benefits of on-demand PrEP suggested that MSM who have poor adherence to oral PrEP can still achieve similar effectiveness in reducing HIV acquisition by using on-demand PrEP [11,17]. Our findings suggest that MSM who have sexual behavioral risks may particularly benefit from on-demand PrEP. Our findings also align with previous studies suggesting that LA PrEP may be a particularly preferable option among MSM at higher risk of HIV acquisition [30]. Additionally, willingness to use LA PrEP was higher among participants who used illicit drugs versus those who did not. In parallel to these findings, acceptance of LA PrEP was previously shown to be high among people who inject drugs [25].

Among current DO PrEP users, a higher willingness to switch to LA PrEP was associated with a lower adherence to the current daily regimen. Participants who did not take the approved number of doses of DO PrEP in the last 30 days (used 15 or less doses or used 16-29 doses) were more willing to switch to LA PrEP compared to those who fully adhered to the DO protocol (30 doses) in the last 30 days. Willingness to switch from DO PrEP may be related to adherence challenges [40,41]. LA PrEP use includes less frequent dosing compared to DO PrEP. DO PrEP users who are aware of their suboptimal adherence might be more willing to switch to LA PrEP [42]. LA PrEP might facilitate increased adherence among MSM and might potentially increase PrEP uptake.

Among current PrEP users, there was also a clear association between willingness to switch to on-demand PrEP and taking fewer doses of DO PrEP in the past 30 days. It is worth noting that out of the 150 individuals who took 15 or less doses within the past 30 days, 42% stated that they only planned to use PrEP during sexual encounters, whereas 10% indicated that they intended to take it on a different schedule. It is possible that some participants might have intended to use PrEP when necessary, even though they were prescribed DO PrEP. Although this strategy is not common among MSM, those who were taking 15 or less doses of DO PrEP might have believed they were already using on-demand PrEP and may have seen a need to switch to an on-demand regimen [16,43]. In our analysis, this could have resulted in a conflation of participants who were DO PrEP users and who were using DO PrEP only when they needed it.

Our findings also suggest that participants residing in rural micropolitan or noncore areas have a higher willingness to switch to on-demand PrEP than those living in large central metro areas. This might be related to challenges in accessing PrEP from rural pharmacies or clinics, making the intermittent use of medication specifically during sexual encounters more appealing than consistently maintaining a supply [44,45]. Also, higher levels of stigma are associated with using HIV prevention medications in rural areas compared to more urban areas [46,47]. The negative perception toward individuals taking PrEP may be higher in rural areas due to the close-knit social dynamics prevalent in small towns. In these areas, people tend to have higher familiarity with each other’s activities, increasing public knowledge of potentially stigmatized behaviors, which can lead to discrimination from various groups, including community members and health care providers [48,49]. HIV and PrEP stigma often act as barriers to PrEP uptake and persistence [10]. Previous research has highlighted that MSM have concerns about the possibility of other individuals, including sexual partners, becoming aware of their PrEP use if they are taking daily regimens [50]. Furthermore, MSM who experienced intimate partner violence, where their behaviors were monitored by their partners, ranked daily oral PrEP lower than other PrEP options [51]. These findings suggest that switching to the LA PrEP option could provide more confidentiality than taking pills daily; this might help to increase PrEP uptake overall.

Prior LA PrEP awareness was another factor associated with a higher willingness to start or switch to LA PrEP options in this study. Willingness to use PrEP was shown to increase with improved PrEP awareness in prior studies including among young MSM [39,52-54]. These results highlight the significance of health education, public education campaigns via social media, and open, informative dialogue between health care providers and patients regarding the newer PrEP options.

We observed regional variations in the PrEP rankings showing that participants living in the West and South rated LA PrEP as their preferred first option for starting PrEP, compared to those in the Northeast. Considering that the highest rates of HIV [55] and the lowest PrEP uptake levels are being reported in the South [2], a high willingness to use LA PrEP in this region may be promising to create opportunities to increase PrEP uptake. However, although these findings offer insights into regional differences in PrEP option preferences, it is important to acknowledge that we were unable to differentiate the disparities between demographically and socioeconomically varied states within these regions which may have provided further insights.

This study has some limitations. Data were collected via online recruitment using a convenience sampling approach; therefore, our results have limited external generalizability. In addition, because we recruited from online sex-seeking apps, our study sample may have a higher proportion of men who may benefit from PrEP [56]. Also, the survey consisted of only self-reported behaviors, so our data are subject to misclassification: it is possible that less socially desirable responses may be underreported by participants even though the survey was anonymous and self-administered [57]. Although we explored urban or rural differences in PrEP preferences, other geographical factors, such as Medicaid expansion status or PrEP provider density could be important determinants in PrEP preferences and warrant investigation in future studies [58,59]. Finally, we asked PrEP preferences of participants in a hypothetical scenario, where PrEP options were available free of charge. However, costs associated with PrEP might create PrEP access barriers in real-world settings.

### Conclusions

Our results highlight the substantial interest among US MSM in starting or switching to on-demand and LA PrEP options. Our findings also indicate that those at higher risk for HIV acquisition or who may be struggling with DO PrEP adherence are substantially more interested in these PrEP options. Increased and varied PrEP options are even more likely to increase overall community PrEP uptake if PrEP options offered by clinicians are informed by understanding differences in preferences among US MSM with different demographic backgrounds, risk profiles, and prior PrEP experiences.

## Appendix

Multimedia Appendix 1 Additional tables.

## Abbreviations

AMIS: The American Men’s Internet Survey
DO: daily oral
LA: long-acting
LGBTQ+: lesbian, gay, bisexual, transgender, queer/questioning, and others
MSM: men who have sex with men
PrEP: preexposure prophylaxis
STI: sexually transmitted infection

## Data Availability

Data cannot be shared publicly because of the sensitive nature of the data but are available from Dr. Travis Sanchez on reasonable request.

## References

[1] Chou R, Spencer H, Bougatsos C, Blazina I, Ahmed A, Selph S (2023). Preexposure prophylaxis for the prevention of HIV: updated evidence report and systematic review for the US preventive services task force. JAMA.

[2] Sullivan PS, Sanchez TH, Zlotorzynska M, Chandler CJ, Sineath RC, Kahle E, Tregear S (2020). National trends in HIV pre-exposure prophylaxis awareness, willingness and use among United States men who have sex with men recruited online, 2013 through 2017. J Int AIDS Soc.

[3] Hudrudchai S, Suwanwong C, Prasittichok P, Mohan KP, Janeaim N (2024). Pre-exposure prophylaxis adherence among men who have sex with men: a systematic review and meta-analysis. J Prev Med Public Health.

[4] Gebru NM, Canidate SS, Liu Y, Schaefer SE, Pavila E, Cook RL, Leeman RF (2023). Substance use and adherence to HIV pre-exposure prophylaxis in studies enrolling men who have sex with men and transgender women: a systematic review. AIDS Behav.

[5] Haberer JE, Mujugira A, Mayer KH (2023). The future of HIV pre-exposure prophylaxis adherence: reducing barriers and increasing opportunities. Lancet HIV.

[6] (2021). FDA approves first injectable treatment for HIV pre-exposure prevention. US Food and Drug Administration.

[7] Landovitz RJ, Donnell D, Clement ME, Hanscom B, Cottle L, Coelho L, Cabello R, Chariyalertsak S, Dunne EF, Frank I, Gallardo-Cartagena JA, Gaur AH, Gonzales P, Tran HV, Hinojosa JC, Kallas EG, Kelley CF, Losso MH, Madruga JV, Middelkoop K, Phanuphak N, Santos B, Sued O, Valencia Huamaní J, Overton ET, Swaminathan S, Del Rio C, Gulick RM, Richardson P, Sullivan P, Piwowar-Manning E, Marzinke M, Hendrix C, Li M, Wang Z, Marrazzo J, Daar E, Asmelash A, Brown TT, Anderson P, Eshleman SH, Bryan M, Blanchette C, Lucas J, Psaros C, Safren S, Sugarman J, Scott H, Eron JJ, Fields SD, Sista ND, Gomez-Feliciano K, Jennings A, Kofron RM, Holtz TH, Shin K, Rooney JF, Smith KY, Spreen W, Margolis D, Rinehart A, Adeyeye A, Cohen MS, McCauley M, Grinsztejn B (2021). Cabotegravir for HIV prevention in cisgender men and transgender women. N Engl J Med.

[8] Fonner VA, Ridgeway K, van der Straten A, Lorenzetti L, Dinh N, Rodolph M, Schaefer R, Schmidt HMA, Nguyen VTT, Radebe M, Peralta H, Baggaley R (2023). Safety and efficacy of long-acting injectable cabotegravir as preexposure prophylaxis to prevent HIV acquisition. AIDS.

[9] Cole SW, Glick JL, Campoamor NB, Sanchez TH, Sarkar S, Vannappagari V, Rinehart A, Rawlings K, Sullivan PS, Bridges JFP (2024). Willingness and preferences for long-acting injectable PrEP among US men who have sex with men: a discrete choice experiment. BMJ Open.

[10] Rosengren AL, Lelutiu-Weinberger C, Woodhouse EW, Sandanapitchai P, Hightow-Weidman LB (2021). A scoping review of HIV pre-exposure prophylaxis stigma and implications for stigma-reduction interventions for men and transwomen who have sex with men. AIDS Behav.

[11] Kwan TH, Lui GCY, Lam TTN, Lee KCK, Wong NS, Chan DPC, Lee SS (2021). Comparison between daily and on-demand PrEP (pre-exposure prophylaxis) regimen in covering condomless anal intercourse for men who have sex with men in Hong Kong: a randomized, controlled, open-label, crossover trial. J Int AIDS Soc.

[12] Molina JM, Capitant C, Spire B, Pialoux G, Cotte L, Charreau I, Tremblay C, Le Gall JM, Cua E, Pasquet A, Raffi F, Pintado C, Chidiac C, Chas J, Charbonneau P, Delaugerre C, Suzan-Monti M, Loze B, Fonsart J, Peytavin G, Cheret A, Timsit J, Girard G, Lorente N, Préau M, Rooney JF, Wainberg MA, Thompson D, Rozenbaum W, Doré V, Marchand L, Simon MC, Etien N, Aboulker JP, Meyer L, Delfraissy JF (2015). On-demand preexposure prophylaxis in men at high risk for HIV-1 infection. N Engl J Med.

[13] Molina JM, Charreau I, Spire B, Cotte L, Chas J, Capitant C, Tremblay C, Rojas-Castro D, Cua E, Pasquet A, Bernaud C, Pintado C, Delaugerre C, Sagaon-Teyssier L, Mestre SL, Chidiac C, Pialoux G, Ponscarme D, Fonsart J, Thompson D, Wainberg MA, Doré V, Meyer L (2017). Efficacy, safety, and effect on sexual behaviour of on-demand pre-exposure prophylaxis for HIV in men who have sex with men: an observational cohort study. Lancet HIV.

[14] Camp C, Saberi P (2021). Facilitators and barriers of 2-1-1 HIV pre-exposure prophylaxis. PLoS One.

[15] Hojilla JC, Marcus JL, Silverberg MJ, Hare CB, Herbers R, Hurley L, Satre DD, Volk JE (2020). Early adopters of event-driven human immunodeficiency virus pre-exposure prophylaxis in a large healthcare system in San Francisco. Clin Infect Dis.

[16] Sewell WC, Powell VE, Mayer KH, Ochoa A, Krakower DS, Marcus JL (2020). Nondaily use of HIV preexposure prophylaxis in a large online survey of primarily men who have sex with men in the United States. J Acquir Immune Defic Syndr.

[17] Stansfield SE, Moore M, Boily MC, Hughes JP, Donnell DJ, Dimitrov DT (2023). Estimating benefits of using on-demand oral prep by MSM: a comparative modeling study of the US and Thailand. EClinicalMedicine.

[18] Schoenberg P, Edwards OW, Merrill L, Martinez CA, Stephenson R, Sullivan PS, Jones J (2023). Willingness to use and preferences for long-acting injectable PrEP among sexual and gender minority populations in the southern United States, 2021-2022: cross-sectional study. J Int AIDS Soc.

[19] Levy ME, Agopian A, Magnus M, Rawls A, Opoku J, Kharfen M, Greenberg AE, Kuo I (2021). Is long-acting injectable cabotegravir likely to expand PrEP coverage among MSM in the District of Columbia?. J Acquir Immune Defic Syndr.

[20] Gordián-Arroyo A, Garofalo R, Kuhns LM, Pearson C, Bruce J, Batey DS, Radix A, Belkind U, Hidalgo MA, Hirshfield S, Schrimshaw EW, Schnall R (2020). Awareness, willingness, and perceived efficacy of pre-exposure prophylaxis among adolescent sexual minority males. J Urban Health.

[21] Weiss KM, Prasad P, Sanchez T, Goodreau SM, Jenness SM (2021). Association between HIV PrEP indications and use in a national sexual network study of US men who have sex with men. J Int AIDS Soc.

[22] Bonacci RA, Van Handel M, Huggins R, Inusah S, Smith DK (2023). Estimated uncovered costs for HIV preexposure prophylaxis in the US, 2018. Health Aff.

[23] Morgan E, Moran K, Ryan DT, Mustanski B, Newcomb ME (2018). Threefold increase in PrEP uptake over time with high adherence among young men who have sex with men in Chicago. AIDS Behav.

[24] Hart TA, Noor SW, Berlin GW, Skakoon-Sparling S, Tavangar F, Tan D, Lambert G, Grace D, Lachowsky NJ, Jollimore J, Sang J, Parlette A, Lal A, Apelian H, Moore D, Cox J (2023). Pre-exposure prophylaxis and bacterial sexually transmitted infections (STIs) among gay and bisexual men. Sex Transm Infect.

[25] King AR, Shah S, Randall LA, Frew PM, Spaulding A, Holloway IW, HBOU Study Team (2022). Acceptability of injectable pre-exposure prophylaxis among people who inject drugs in three urban U.S. settings. BMC Infect Dis.

[26] Sanchez TH, Sineath RC, Kahle EM, Tregear SJ, Sullivan PS (2015). The annual American Men's Internet Survey of behaviors of men who have sex with men in the United States: protocol and key indicators report 2013. JMIR Public Health Surveill.

[27] Sanchez TH, Zlotorzynska M, Sineath RC, Kahle E, Tregear S, Sullivan PS (2018). National trends in sexual behavior, substance use and HIV testing among United States men who have sex with men recruited online, 2013 through 2017. AIDS Behav.

[28] Ingram D, Franco SJ (2014). 2013 NCHS urban-rural classification scheme for counties. Vital Health Stat 2.

[29] John SA, Whitfield THF, Rendina HJ, Parsons JT, Grov C (2018). Will gay and bisexual men taking oral pre-exposure prophylaxis (PrEP) switch to long-acting injectable PrEP should it become available?. AIDS Behav.

[30] Biello KB, Mimiaga MJ, Santostefano CM, Novak DS, Mayer KH (2018). MSM at highest risk for HIV acquisition express greatest interest and preference for injectable antiretroviral PrEP compared to daily, oral medication. AIDS Behav.

[31] Levy ME, Patrick R, Gamble J, Rawls A, Opoku J, Magnus M, Kharfen M, Greenberg AE, Kuo I (2017). Willingness of community-recruited men who have sex with men in Washington, DC to use long-acting injectable HIV pre-exposure prophylaxis. PLoS One.

[32] Mansergh G, Kota KK, Stephenson R, Hirshfield S, Sullivan P (2021). Preference for using a variety of future HIV pre-exposure prophylaxis products among men who have sex with men in three US cities. J Int AIDS Soc.

[33] García M, Harris AL (2017). PrEP awareness and decision-making for Latino MSM in San Antonio, Texas. PLoS One.

[34] Kamitani E, Wichser ME, Mizuno Y, DeLuca JB, Higa DH (2023). What factors are associated with willingness to use HIV pre-exposure prophylaxis (PrEP) among U.S. men who have sex with men not on PrEP? A systematic review and meta-analysis. J Assoc Nurses AIDS Care.

[35] Kirby T (2020). PrEP use falling short in African American and Hispanic MSM. Lancet HIV.

[36] Riley T, Anaya G, Gallegos PA, Castaneda R, Khosropour CM (2023). Pre-exposure prophylaxis use and discontinuation in a federally qualified health center in a Mexico-US border city. J Racial Ethn Health Disparities.

[37] Hosek SG, Siberry G, Bell M, Lally M, Kapogiannis B, Green K, Fernandez MI, Rutledge B, Martinez J, Garofalo R, Wilson CM (2013). The acceptability and feasibility of an HIV preexposure prophylaxis (PrEP) trial with young men who have sex with men. J Acquir Immune Defic Syndr.

[38] Brooks RA, Kaplan RL, Lieber E, Landovitz RJ, Lee S, Leibowitz AA (2011). Motivators, concerns, and barriers to adoption of preexposure prophylaxis for HIV prevention among gay and bisexual men in HIV-serodiscordant male relationships. AIDS Care.

[39] John SA, Zapata JP, Dang M, Pleuhs B, O'Neil A, Hirshfield S, Walsh JL, Petroll AE, Quinn KG (2023). Exploring preferences and decision-making about long-acting injectable HIV pre-exposure prophylaxis (PrEP) among young sexual minority men 17-24 years old. Sci Rep.

[40] Sidebottom D, Ekström AM, Strömdahl S (2018). A systematic review of adherence to oral pre-exposure prophylaxis for HIV - how can we improve uptake and adherence?. BMC Infect Dis.

[41] Whiteley L, Craker L, Sun S, Tarantino N, Hershkowitz D, Moskowitz J, Arnold T, Haubrick K, Olsen E, Mena L, Brown LK (2021). Factors associated with PrEP adherence among MSM living in Jackson, Mississippi. J HIV AIDS Soc Serv.

[42] Rogers BG, Chan PA, Sutten-Coats C, Zanowick-Marr A, Patel RR, Mena L, Goedel WC, Chu C, Silva E, Galipeau D, Arnold T, Gomillia C, Curoe K, Villalobos J, Underwood A, Sosnowy C, Nunn AS (2023). Perspectives on long-acting formulations of pre-exposure prophylaxis (PrEP) among men who have sex with men who are non-adherent to daily oral PrEP in the United States. BMC Public Health.

[43] Caba AE, Rathus T, Burson E, Chan PA, Eaton LA, Watson RJ (2022). Who is using PrEP on-demand? Factors associated with PrEP use modality among black and hispanic/latino emerging adults. AIDS Behav.

[44] Siegler AJ, Bratcher A, Weiss KM (2019). Geographic access to preexposure prophylaxis clinics among men who have sex with men in the United States. Am J Public Health.

[45] Zhao A, Dangerfield DT, Nunn A, Patel R, Farley JE, Ugoji CC, Dean LT (2022). Pharmacy based interventions to increase use of HIV pre-exposure prophylaxis in the United States: a scoping review. AIDS Behav.

[46] Walters SM, Frank D, Van Ham B, Jaiswal J, Muncan B, Earnshaw V, Schneider J, Friedman SR, Ompad DC (2022). PrEP care continuum engagement among persons who inject drugs: rural and urban differences in stigma and social infrastructure. AIDS Behav.

[47] Owens C, Hubach RD, Williams D, Voorheis E, Lester J, Reece M, Dodge B (2020). Facilitators and barriers of pre-exposure prophylaxis (PrEP) uptake among rural men who have sex with men living in the midwestern U.S. Arch Sex Behav.

[48] Ezell JM, Walters S, Friedman SR, Bolinski R, Jenkins WD, Schneider J, Link B, Pho MT (2021). Stigmatize the use, not the user? Attitudes on opioid use, drug injection, treatment, and overdose prevention in rural communities. Soc Sci Med.

[49] Carter G, Meyerson B, Rivers P, Crosby R, Lawrence C, Cope SD, DeBruicker D, Levin S, Meeks W, Thomas C, Turner B, Abert C, Coles H, Allen A, Gonzalez-Fagoaga E, Grivois-Shah R (2021). Living at the confluence of stigmas: PrEP awareness and feasibility among people who inject drugs in two predominantly rural states. AIDS Behav.

[50] Franks J, Hirsch-Moverman Y, Loquere AS, Amico KR, Grant RM, Dye BJ, Rivera Y, Gamboa R, Mannheimer SB (2018). Sex, PrEP, and stigma: experiences with HIV pre-exposure prophylaxis among New York City MSM participating in the HPTN 067/ADAPT study. AIDS Behav.

[51] Stephenson R, Rogers E, Mansergh G, Hirshfield S, Sullivan P (2022). Intimate partner violence and preferences for pre-exposure prophylaxis (PrEP) modes of delivery among a sample of gay, bisexual, and other men who have sex with men. AIDS Behav.

[52] Sun Z, Gu Q, Dai Y, Zou H, Agins B, Chen Q, Li P, Shen J, Yang Y, Jiang H (2022). Increasing awareness of HIV pre-exposure prophylaxis (PrEP) and willingness to use HIV PrEP among men who have sex with men: a systematic review and meta-analysis of global data. J Int AIDS Soc.

[53] Taggart T, Liang Y, Pina P, Albritton T (2020). Awareness of and willingness to use PrEP among black and latinx adolescents residing in higher prevalence areas in the United States. PLoS One.

[54] Shrestha R, DiDomizio EE, Kim RS, Altice FL, Wickersham JA, Copenhaver MM (2020). Awareness about and willingness to use long-acting injectable pre-exposure prophylaxis (LAI-PrEP) among people who use drugs. J Subst Abuse Treat.

[55] Centers for Disease Control and Prevention (2021). HIV surveillance report: Diagnoses of HIV infection in the United States and dependent areas, 2021.

[56] Wiatrek S, Zlotorzynska M, Rai R, Sullivan P, Sanchez T (2021). The annual American Men's Internet Survey of behaviors of men who have sex with men in the United States: key indicators report 2018. JMIR Public Health Surveill.

[57] Gnambs T, Kaspar K (2015). Disclosure of sensitive behaviors across self-administered survey modes: a meta-analysis. Behav Res Methods.

[58] Farkhad BF, Holtgrave DR, Albarracín D (2021). Effect of Medicaid expansions on HIV diagnoses and pre-exposure prophylaxis use. Am J Prev Med.

[59] Kim B, Chaix B, Chen YT, Callander D, Regan SD, Duncan DT (2021). Geographic density and uptake of pre-exposure prophylaxis (PrEP) among young gay, bisexual and other sexual minority men: a global positioning system (GPS) study. AIDS Behav.

