# Supplementary material for: Preferences for Starting Daily, On-Demand, and Long-Acting Injectable HIV Preexposure Prophylaxis Among Men Who Have Sex With Men in the United States (2021-2022): Nationwide Online Cross-Sectional Study
Source: JMIR Public Health Surveill. 2024 Nov 13;10:e62801. doi: 10.2196/62801 (PMC11602762; doi:10.2196/62801)
Supplement: Multimedia Appendix 1 [file publichealth_v10i1e62801_app1.docx]

**Table S1.** Brief descriptions of each PrEP modality given to participants.

| LA-PrEP | A long-acting form of PrEP that is given as an injection in your buttocks once every 2 months has been found to be effective in preventing HIV infection. You would have to see a doctor or healthcare provider to start injectable PrEP and go back for check-ups once every 2 months to stay on it. The possible side effects of injectable PrEP are mild-to-moderate pain at the injection site that lasts 2 to 7 days, mild rash at the injection site that clears up on its own, or a headache that lasts a couple of days after injection. Studies have found that injectable PrEP is effective at preventing HIV infection. This approach isn’t currently approved by the FDA (Food and Drug Administration). |
| --- | --- |
| Daily Oral PrEP | PrEP stands for pre-exposure prophylaxis. Daily oral PrEP is a pill that a person who is HIV-negative takes every day in order to prevent getting HIV. PrEP is safe, but some people experience side effects like diarrhea, nausea, headache, fatigue, and stomach pain. These side effects usually go away over time. You have to see a doctor or other healthcare provider to start daily oral PrEP and go back for check-ups once every 3 months to stay on it. Studies have shown that daily oral PrEP provides about 99% protection against HIV infection when it is taken every day |
| On-demand Oral PrEP | There is another way that some people take PrEP pills called on-demand oral PrEP. On-demand oral PrEP is also known as “intermittent” or “event-driven” PrEP. With on-demand oral PrEP you take a series of pills around the time when you have sex. This means taking two pills 2 to 24 hours before sex, one pill 24 hours after the first dose, and one pill 24 hours after the second dose. Some studies found that on-demand PrEP is effective at preventing HIV infection. This approach isn’t currently approved by the FDA (Food and Drug Administration). |

**Table S2.** Characteristics of the analytic sample, AMIS 2021-22 (N=7760).

|  | **n** | **%** |
| --- | --- | --- |
| Total | 7760 | 100 |
| **Age (years)** |  |  |
| 15-24 | 707 | 9.1 |
| 25-29 | 847 | 10.9 |
| 30-39 | 1957 | 25.2 |
| 40+ | 4249 | 54.8 |
| **Race/Ethnicity** |  |  |
| Black, non-Hispanic/Latino | 784 | 10.2 |
| Hispanic or Latino | 1086 | 14.1 |
| White, non-Hispanic/Latino | 5114 | 66.6 |
| Other or multiple races | 698 | 9.1 |
| **Health insurance** |  |  |
| None | 521 | 6.8 |
| Private only | 5459 | 71.6 |
| Public only | 1191 | 15.6 |
| Other or multiple insurance | 452 | 5.9 |
| **Education level** |  |  |
| < HS diploma | 128 | 1.7 |
| HS diploma or equivalent | 705 | 9.1 |
| Some college or technical degree | 1959 | 25.3 |
| College degree or postgraduate education | 4945 | 63.9 |
| **Employment status** |  |  |
| Not employed | 472 | 6.1 |
| Employed for wages full-time | 5073 | 65.9 |
| Employed for wages part-time | 609 | 7.9 |
| Self employed | 711 | 9.2 |
| A homemaker | 26 | 0.3 |
| Retired | 675 | 8.8 |
| Unable to work(disabled) | 134 | 1.7 |
| **Household Income** |  |  |
| $0-19999 | 628 | 8.6 |
| $20000-39999 | 1128 | 15.5 |
| $40000-74999 | 1697 | 23.3 |
| $75000 or more | 3825 | 52.6 |
| **NCHS urban-rural category** | |  |
| Large central metro | 3467 | 44.8 |
| Large fringe metro | 1630 | 21.1 |
| Medium metro | 1463 | 18.9 |
| Small metro | 560 | 7.2 |
| Micropolitan | 401 | 5.2 |
| Non-core | 220 | 2.8 |
| **Census region** |  |  |
| Northeast | 1465 | 18.9 |
| Midwest | 1548 | 20.0 |
| South | 2931 | 37.8 |
| West | 1804 | 23.3 |
| U.S. dependent areas | 12 | 0.2 |
| **STI diagnosis in in past 12 months** |  |  |
| No | 6864 | 88.5 |
| Yes | 896 | 11.6 |
| **Condomless anal sex in past 12 months** | |  |
| No | 1986 | 25.6 |
| Yes | 5774 | 74.4 |
| **Number of male sex partners** | |  |
| One | 1514 | 19.5 |
| 2 or more | 6061 | 78.1 |
| **Any illicit drug use in past 12 months** | |  |
| No | 5481 | 70.6 |
| Yes | 2279 | 29.4 |
| **Used PrEP in past 12 months** |  |  |
| No | 5108 | 65.8 |
| Yes | 2652 | 34.2 |

*Abbreviations: NCHS: National Center for Health Statistics, NHBS; National HIV Behavioral Surveillance,

STI: Sexually Transmitted Infections, PrEP :Pre-exposure prophylaxis

**Table S3.** Characteristics associated with ranking long-acting injectable PrEP as a first preference to start PrEP versus daily oral or on-demand PrEP among men who have sex with men and who did not use PrEP in past twelve months, American Men’s Internet Survey, 2021-22.

|  | **Rank LA PrEP as a first option versus daily oral or on-demand PrEP** | | **PR and 95% CI** | **Adjusted PR and 95% CI*** |
| --- | --- | --- | --- | --- |
|  | **Yes**  **n (%)** | **No**  **n (%)** |  |  |
| Total | 659 (48.2) | 709 (51.8) |  |  |
| **Age (years)** |  |  |  |  |
| 15-24 | 69 (42.1) | 95 (57.9) | 0.87 (0.72 , 1.06) | 0.86 (0.71 , 1.04) |
| 25-29 | 67 (44.7) | 83 (55.3) | 0.93 (0.76 , 1.12) | 0.91 (0.74 , 1.11) |
| 30-39 | 184 (52.4) | 167 (47.6) | 1.09 (0.96 , 1.23) | 1.06 (0.93 , 1.21) |
| 40+ | 339 (48.2) | 364 (51.8) | ref | ref |
| **Race/Ethnicity** |  |  |  |  |
| Black, non-Hispanic/Latino | 65 (47.8) | 71 (52.2) | 0.97 (0.80 , 1.17) | 1.03 (0.85 , 1.25) |
| Hispanic or Latino | 113 (44.3) | 142 (55.7) | 0.90 (0.77 , 1.05) | 0.86 (0.73 , 1.01) |
| White, non-Hispanic/Latino | 420 (49.2) | 433 (50.8) | ref | ref |
| Other or multipl races | 55 (48.7) | 58 (51.3) | 0.99 (0.81 , 1.21) | 0.93 (0.76 , 1.15) |
| **Health insurance** |  |  |  |  |
| None | 58 (41.4) | 82 (58.6) | 0.81 (0.66 , 1.00) | 0.85 (0.69 , 1.05) |
| Private only | 466 (51.1) | 446 (48.9) | ref | ref |
| Public only | 98 (43.9) | 125 (56.1) | 0.86 (0.73 , 1.01) | 0.88 (0.75 , 1.04) |
| Other or multiple insurance | 26 (38.8) | 41 (61.2) | 0.76 (0.56 , 1.03) | 0.69 (0.51 , 0.94) |
| **NCHS urban-rural category** |  |  |  |  |
| Large central metro | 264 (51.8) | 246 (48.2) | ref | ref |
| Large fringe metro | 126 (42.7) | 169 (57.3) | 0.83 (0.71 , 0.96) | 0.84 (0.72 , 0.99) |
| Medium metro | 147 (48.2) | 158 (51.8) | 0.93 (0.81 , 1.07) | 0.91 (0.77 , 1.08) |
| Small metro | 52 (43.7) | 67 (56.3) | 0.84 (0.68 , 1.05) | 0.86 (0.68 , 1.08) |
| Micropolitan and non-core | 66 (50.0) | 66 (50.0) | 0.97 (0.80 , 1.17) | 0.97 (0.79 , 1.19) |
| **Census region** |  |  |  |  |
| Northeast | 110 (44.9) | 135 (55.1) | ref | ref |
| Midwest | 113 (44.5) | 141 (55.5) | 0.99 (0.82 , 1.20) | 0.98 (0.80 , 1.21) |
| South | 260 (46.5) | 299 (53.5) | 1.04 (0.88 , 1.22) | 1.04 (0.88 , 1.24) |
| West | 174 (56.1) | 133 (42.9) | 1.26 (1.07 , 1.50) | 1.27 (1.07 , 1.52) |
| **STI diagnosis in past 12 months** |  |  |  |  |
| No | 602 (47.6) | 662 (52.4) | ref | ref |
| Yes | 57 (54.8) | 47 (45.2) | 1.15 (0.96 , 1.38) | 1.18 (0.99 , 1.42) |
| **Condomless anal sex in past 12 months** |  |  |  |  |
| No | 169 (45.9) | 199 (54.1) | ref | ref |
| Yes | 490 (49.0) | 510 (51.0) | 1.07 (0.94 , 1.21) | 1.02 (0.89 , 1.16) |
| **Number of male sex partners** |  |  |  |  |
| One | 107 (47.1) | 120 (52.9) | ref | ref |
| 2 or more | 540 (48.6) | 572 (51.4) | 1.03 (0.89 , 1.20) | 1.00 (0.86 , 1.16) |
| **Any illicit drug use past 12 months** |  |  |  |  |
| No | 446 (45.5) | 534 (54.5) | ref | ref |
| Yes | 213 (54.9) | 175 (45.1) | 1.21 (1.08 , 1.35) | 1.18 (1.05 , 1.33) |
| **Prior awareness of LA PrEP** |  |  |  |  |
| No | 512 (46.4) | 592 (53.6) | 1.22 (1.07 , 1.38) | 1.13 (0.99 , 1.28) |
| Yes | 146 (56.4) | 113 (43.6) | ref | ref |

*Abbreviations: NCHS: National Center for Health Statistics, NHBS; National HIV Behavioral Surveillance,

STI: Sexually Transmitted Infections, PrEP : Pre-exposure prophylaxis, PR: Prevalence Ratio

**Table S4**. Characteristics associated with ranking long-acting injectable PrEP as a first preference to switch from daily oral PrEP versus switching to on-demand PrEP among men who have sex with men and who are current PrEP users, American Men’s Internet Survey, 2021-22.

|  | **Rank LA PrEP as a first option for switching versus on-demand PrEP** | | **PR (95 % CI)** |
| --- | --- | --- | --- |
|  | **Yes (n %)** | **No (n %)** |  |
| Total | 342 (69.7 ) | 149 (30.3) |  |
| **Age (years)** |  |  |  |
| 15-24 | 14 (70.0) | 6 (30.0) | 1.04 (0.77 , 1.40) |
| 25-29 | 32 (69.6) | 14 (30.4) | 1.03 (0.84 , 1.27) |
| 30-39 | 104 (74.3) | 36 (25.7) | 1.10 (0.97 , 1.25) |
| 40+ | 192 (67.4) | 93 (32.6) | ref |
| **Race/Ethnicity** |  |  |  |
| Black, non-Hispanic/Latino | 34 (72.3) | 13 (27.7) | 1.09 (0.89 , 1.32) |
| Hispanic or Latino | 61 (74.4) | 21 (25.6) | 1.12 (0.96 , 1.30) |
| White, non-Hispanic/Latino | 208 (66.7) | 104 (33.3) | ref |
| Other or multiple races | 35 (79.5) | 9 (20.5) | 1.19 (1.01 , 1.41) |
| **Health insurance** |  |  |  |
| None | 22 (81.5) | 5 (18.5) | 1.16 (0.96 , 1.41) |
| Private only | 263 (70.1) | 112 (29.9) | ref |
| Public only | 46 (67.6) | 22 (32.4) | 0.96 (0.81 , 1.15) |
| Other or multiple insurance | 9 (50.0) | 9 (50.0) | 0.71 (0.45 , 1.14) |
| **NCHS urban-rural category** |  |  |  |
| Large central metro | 179 (70.2) | 76 (29.8) | ref |
| Large fringe metro | 67 (67.7) | 32 (32.3) | 0.96 (0.82 , 1.13) |
| Medium metro | 52 (66.7) | 26 (33.3) | 0.95 (0.80 , 1.13) |
| Small metro | 23 (74.2) | 8 (25.8) | 1.06 (0.85 , 1.32) |
| Micropolitan and non-core | 21 (75.0) | 7 (25.0) | 1.07 (0.85 , 1.34) |
| **Census region** |  |  |  |
| Northeast | 65 (70.7) | 27 (29.3) | ref |
| Midwest | 53 (66.3) | 27 (33.8) | 0.94 (0.76 , 1.15) |
| South | 139 (73.9) | 49 (26.1) | 1.05 (0.89 , 1.22) |
| West | 85 (64.9) | 46 (35.1) | 0.92 (0.77 , 1.10) |
| **STI diagnosis in past 12 months** |  |  |  |
| No | 263 (68.7) | 120 (31.3) | ref |
| Yes | 79 (73.1) | 29 (26.9) | 1.07 (0.93 , 1.22) |
| **Condomless anal sex in past 12 months** |  |  |  |
| No | 44 (66.7) | 22 (33.3) | ref |
| Yes | 298 (70.1) | 127 (29.9) | 1.05 (0.88 , 1.26) |
| **Number of male sex partners** |  |  |  |
| One | 12 (50.0) | 12 (50.0) | ref |
| 2 or more | 320 (70.6) | 133 (29.4) | 1.41 (0.94 , 2.12) |
| **Any illicit drug use past 12 months** |  |  |  |
| No | 198 (66.0) | 102 (34.0) | ref |
| Yes | 144 (75.4) | 47 (24.6) | 1.14 (1.02 , 1.28) |
| **Prior awareness of LA PrEP** |  |  |  |
| No | 230 (66.3) | 117 (33.7) | 1.18 (1.05 , 1.32) |
| Yes | 112 (78.3) | 31 (21.7) | ref |
| **Current PrEP prescription medication** |  |  |  |
| Truvada | 184 (70.0) | 79 (30.0) | ref |
| Descovy | 150 (68.2) | 70 (31.8) | 0.97 (0.86 , 1.10) |
| **Number of PrEP doses taken in last 30 days** |  |  |  |
| <15 | 44 (72.1) | 17 (27.9) | 1.05 (0.88 , 1.25) |
| 16-29 | 82 (70.7) | 34 (29.3) | 1.03 (0.90 , 1.18) |
| 30 | 208 (68.6) | 95 (31.4) | ref |
| **PrEP duration** |  |  |  |
| Less than 2 months | 34 (68.0) | 16 (32.0) | 0.93 (0.76 , 1.14) |
| 2 to 6 months | 47 (62.7) | 28 (37.3) | 0.86 (0.71 , 1.04) |
| 7 to 12 months | 41 (65.1) | 22 (34.9) | 0.89 (0.74 , 1.09) |
| 12 months or more | 219 (72.8) | 82 (27.2) | ref |

*Abbreviations: NCHS: National Center for Health Statistics, NHBS; National HIV Behavioral Surveillance,

STI: Sexually Transmitted Infections, PrEP :Pre-exposure prophylaxis; PR: Prevalence Ratio
